# Deceptive pollinator lures benefit from physical and perceptual proximity to flowers

**DOI:** 10.1002/ece3.11120

**Published:** 2024-03-06

**Authors:** Thomas E. White

**Affiliations:** ^1^ School of Life and Environmental Sciences The University of Sydney Sydney New South Wales Australia

**Keywords:** communication, deception, mimicry, predator–prey, spider

## Abstract

Predators often use deception to exploit sensory and cognitive biases in prey. In pollinating insects, these include preferences for conspicuous colours associated with flowers, which predators such as orb‐web spiders display as prey lures. Theory predicts that deceptive signal efficacy should covary with both their perceptual similarity and physical proximity to the resources—here, flowers—whose cues they are imitating. Here I used the colour‐polymorphic jewelled spider *Gasteracantha fornicata* to test this prediction. I first examined spiders' capture success in the field, and found their visual resemblance and physical proximity to flowers interacted to mediate capture rates, with colour‐similarity becoming increasingly important as the distance between spiders and flowers decreased. I then replicated this interaction experimentally. Spiders adjacent to colour‐matched flowers enjoyed heightened capture success relative to those with nearby but colour‐mismatched flowers. While spiders with flowers placed at a distance (irrespective of colour) recorded the fewest captures. These results support ‘neighbourhood’ effects in aggressive deception as receivers' vulnerability to exploitation is mediated by the local signalling community. More generally, they emphasise the importance of the broader information landscape in the ecology of communication, and suggest misinformation is most effective when physically and perceptually proximate to the truth.

## Graphical abstract

The jewelled spider *Gasteracantha fornicata* uses conspicuous colours, resembling flowers, to lure prey. I show that their capture success is greatest when spiders are positioned near the flowers which they mimic. Misinformation is thus most effective when physically and perceptually proximate to the ‘truth’.

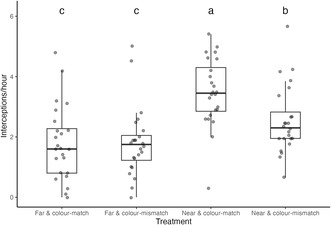

## 1 INTRODUCTION

The natural world is awash with misinformation. Stickleback fish sport false eyes to deflect attacks (Kjernsmo & Merilaita, 2013), cephalopods change colour to disrupt their body outline (Hanlon et al., 2009), and larval moths masquerade as the branches on which they rest (Rowland et al., 2020). Just as deception may be used in defence, so too can it be an instrument of aggression. In general terms, sensory and cognitive systems have evolved in response to a suite of competing demands—including speed, accuracy, efficiency, and performance—which trade off against one another (Chittka et al., 2009; Del Giudice & Crespi, 2018). This produces cognitive and sensory biases in prey, which predators exploit (White et al., 2022; White & Kemp, 2015). Common targets include prey preferences for colours (Schaefer & Ruxton, 2008) or shapes (Gaskett, 2012) which are otherwise a guide to mating or food resources. More elaborate deceptions may instead target higher‐level cognitive processes. The orchid mantis offers a striking example; they combine pastel colouration and flattened, petal‐like limbs in a compelling simulacrum of a flower, and foraging pollinators misclassify them as such (O'Hanlon, 2014).

The effectiveness of deceptive signals is predicted to depend, in part, on the accuracy with which they target said biases in receivers (Christy, 1995; Mokkonen & Lindstedt, 2016). ‘Accuracy’ in deception, however, is often ecologically contextual, particularly when the channels being exploited are as general‐purpose and plastic as colour preferences (White & Kemp, 2015). Colour informs daily decision‐making and acts a guide to essential resources across many animal taxa (Osorio & Vorobyev, 2008). Chromatic cues are also readily learned in association with rewards, as exemplified by the rich gamut of floral advertisements which signal their reward to diverse pollinators (Chittka & Menzel, 1992). The ubiquity of this mutualism renders it a profitable target for aggressive deception, and the colour‐based exploitation of pollinators is well documented (Kemp et al., 2022; O'Hanlon, Holwell, & Herberstein, 2014; Tso et al., 2006). Orb‐web spiders are particularly adept, with some employing the colour, pattern, and/or shape cues associated with flowers to attract the attention of pollinating insects (Tso et al., 2004, 2006; White & Kemp, 2020). They are also often sympatric with species of plants whose floral cues they present, which observational work suggests is necessary to target their shared pool of receivers (White et al., 2017; but see Vieira et al., 2017). These two axes—physical proximity and resemblance—are therefore key to defining the context in which deceptive signals operate, and so should mediate their effectiveness.

The combined importance of physical and sensory proximity between deceptive and honest signallers is well supported in human domains. In marketing, for example, the attention paid to objectively inferior products increases with spatial proximity and physical resemblance to higher‐quality ‘targets’ (Huang., 2021). To state it in more general terms; misinformation is most effective when it is both physically and perceptually proximate to the truth. It stands to reason that a similar dynamic may hold in natural systems, and work in mimetic contexts has partly borne this out. The protective value of much (but not all; McLean et al., 2019) Batesian mimicry is improved by greater resemblance to defended models (Mappes & Alatalo, 1997), and increased range overlap between models and mimics (Pfennig et al., 2001). Similarly, visitations to specialist deceptive orchids is predicted by the fidelity of their mimicry (Benitez‐Vieyra et al., 2007; Peter & Johnson, 2008), and their proximity to models (Peter & Johnson, 2008). Whether these effects extend directly to aggressive contexts, however, is unclear. The general effectiveness of deceptive lures in key systems—such as orb spiders—has long been appreciated (reviewed in Ximenes et al., 2020), but the mechanistic basis of exploitation has proven difficult to test; not least because the receivers being exploited are often more diverse, their biases more general, and putative ‘models’ more unpredictable in appearance and distribution than in traditional model‐mimic systems (White & Kemp, 2015).

The jewelled spider *Gasteracantha fornicata* is an orb‐web spider which inhabits the rainforests of North‐East Queensland, Australia. Females are colour‐polymorphic, and their striking yellow‐ or white—banded dorsal patterns are deceptive lures which chiefly attract flower‐visiting insects (Hauber, 2002; White & Kemp, 2017). Their ventrum, by contrast, bears a more subtle, mottled arrangement of lightly pigmented points, the colour of which matches the dorsal bands. Accumulating evidence paints a broad picture of prey exploitation via floral mimicry in this system, which includes the targeting of innate and (probable) learned attraction to visually salient cues (White & Kemp, 2016a, 2016b), as well as the misclassification of the spiders' signals as flowers (White et al., 2017; White & Kemp, 2020). The shared pool of viewers between deceptive spiders and rewarding flowers, and their targeting of common perceptual channels, makes them an ideal model for understanding the mediators of effective deception in the wild.

Here I used *G. fornicata* to test the extent to which perceptual and physical proximity to ‘models’ shapes the effectiveness of a deceptive signal. I did so in two stages. First, I used an observational assay to estimate how perceptual and spatial proximity between rewarding flowers and deceptive spiders shapes their capture success. Second, I manipulated both axes of proximity in a factorial design, to quantify the causal relationship between each and deceptive signal efficacy. Across both assays the central prediction from theory, as outlined above, is that misinformation should be increasingly effective (hence, capture rates should positively scale) with reduced spatial and perceptual distance between deceptive signallers and models.

## 2 METHODS

I conducted experiments with wild populations of *G. fornicata* in Cairns, Queensland, Australia; in September 2017 for the observational assay, and December 2018 for the manipulative experiment. Both observational and experimental facets took place surrounding a curated botanic garden (−16.899 S, 145.747 E), which is host to a diversity of both native and non‐native angiosperms.

### 2.1 Observing physical and perceptual proximity

I estimated the physical proximity of luring spiders to flowers by measuring, to the nearest mm, the straight‐line distance (i.e. Euclidean) between a given female *G. fornicata* resting in their web to the centre of the nearest inflorescence of any flowering plant. For tractability I excluded any spider whose web was situated over five metres from any flower prior to observation, though only two individuals met this cutoff in the sampled area and were consequently excluded.

**FIGURE 1:**
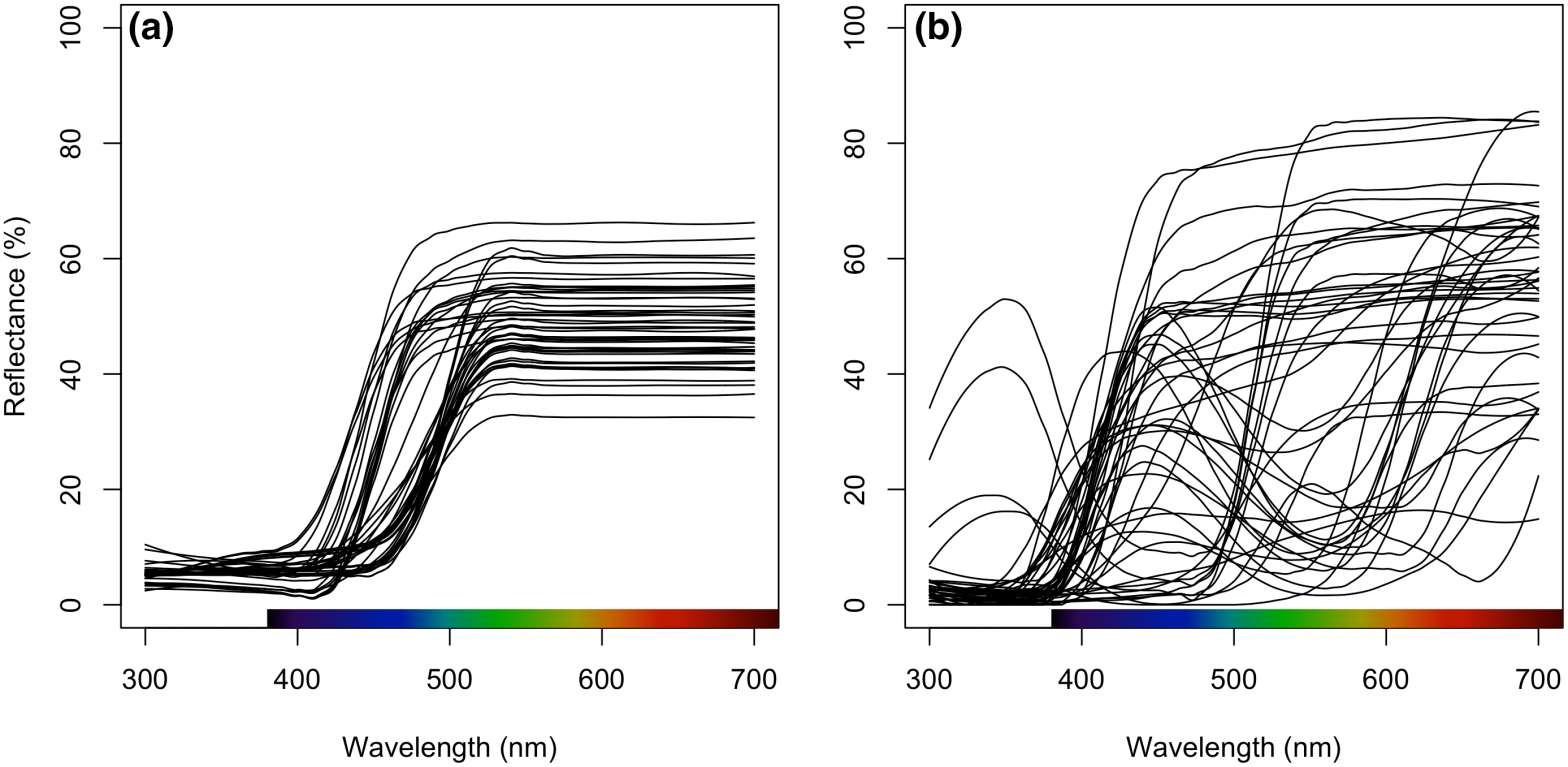
Reflectance spectra of (a) the banded dorsa of female jewelled spiders *Gasteracantha fornicata*, whose conspicuous white‐ or yellow‐and‐black colours function as deceptive prey lures, and (b) the inflorescences which individual spiders were residing nearest to during the observational assay.

I quantified the ‘perceptual’ proximity of spider/flower pairs by first recording the spectral reflectance of each across the UV–visible range (300–700 nm) using an OceanView JAZ portable spectrophotometer, fitted with a 400 μm diameter bifurcated probe with a custom fixed‐angle 45° tip. Following the conclusion of observational transects (below), I removed each spider from its web, gently restrained it in a small foam block cut to size, and recorded two reflectance measurements (subsequently averaged) from either side of their dorsal mid‐line on the central coloured band. I then returned each spider to its web, and observed no obvious ill effects of this brief (ca. 2 min) bout of handling, with many returning to their resting position in the central hub within minutes. I recorded the reflectance of each flower in situ using the same basic method, though here I took two reflectance measurements from the dominant colour (by area) of the adaxial surface of a perianth or showy bract, or occasionally large display stamens. I calibrated the spectrometer against a 99% diffuse white standard (Labsphere, North Sutton, New Hampshire) and dark standard (the occluded spectrometer input) between each individual spider or flower. I lightly LOESS smoothed all spectra (span = 0.15) and zeroed spurious negative values before averaging replicate measures for analysis (Figure 1).

Following spectral recording, I took as my estimate of perceptual proximity the colour distance between each spider and its nearest flower. I used the dipteran colourspace of Troje (1993) with a D65 daylight illuminant, and the visual phenotype of *Drosophila melanogaster* (Sharkey et al., 2020) since flies comprise the vast majority of prey among *G. fornicata*, as validated, in part, at the current study site (White & Kemp, 2016a, 2016b). The broad purpose of such a model is to estimate the ‘colour distance’ (as a measure of difference/similarity), between two stimuli in a morphospace which is defined by the basic structure of the visual system of a relevant viewer—here, a fly. The specific model I employed assumes the involvement of all four dipteran photoreceptor classes, with the vertices of the resulting colourspace defined by two opponent mechanisms (R7p–R8p, and R7y–R8y). I disregarded the original assumption of categorical colour processing (Troje, 1993), however, and instead used the Euclidean distance between points as a continuous measure of colour‐similarity, given more recent and robust evidence in support of this view (Hannah et al., 2019). Like most such models it also represents only early‐stage (i.e. receptor‐level) processing (Kemp et al., 2015), and so does not incorporate downstream effects such as the intersection of chromatic and achromatic circuits, the perceptual consequences of which in *Drosophila* remain to be fully understood (Schnaitmann et al., 2020). I conducted all visual modelling and spectral processing using the packages ‘pavo’ (v2.8.0) and ‘lightr’ (v1.7.0) for R (Gruson et al., 2019; Maia et al., 2019; Maia & White, 2018).

Across both observational and experimental assays the outcome measure of interest was the rate of prey interceptions, as a measure of one key facet of individual fitness. To estimate this I used a walking transect‐based method which has been previously validated against continuous observation in this system (White, 2017; White & Kemp, 2016a, 2016b). Briefly, I recorded the presence of new prey and/or damage to webs at 30 minute intervals over 4 h in the mid to late morning (0900–1200), from which I calculated an hourly interception rate for each individual. This sampling period and timeframe is preferable as it minimises the risk of missed interceptions due to the rapid processing of prey and/or web repair. It also minimises the impact of abiotic confounds such as web damage by debris, which tend to accumulate as the day progresses, though which ultimately contribute only residual variation given the randomisation of treatments among webs (in the experimental assay). This method also cannot determine which side of the web prey approached, and hence whether they viewed the dorsum and/or ventrum of a spider prior to capture. Since the focus of this study is on the colour (or ‘hue’) of polymorphic spiders which consistent in its appearance between dorsal and ventral surfaces of a given morph, rather than their pattern (which does vary, as described above), this too should contribute only residual variation to any identified effects.

### 2.2 Manipulating physical and perceptual proximity

I sought to manipulate the physical and perceptual proximity of spiders to flowers in a complementary test of the causal relationship between each factor and prey capture success. To achieve this I leveraged the fact that female *G. fornicata* are discretely colour polymorphic, with equal prey capture success and composition between morphs (Kemp et al., 2013; White & Kemp, 2016a, 2016b), which allowed me to control colour‐ and physical‐proximity to flowers in a 2 × 2 factorial design. To manipulate perceptual distance, I attached three fresh inflorescences of the perennial shrub *Hibbertia linearis* to the end of a length of wooden dowel (3 m length × 10 mm diameter). The species has been previously identified as a near‐optimal spectral match for the yellow morph of *G. fornicata* (White et al., 2017), which I reaffirmed via reflectance measurement and visual modelling, using the methods described above. When paired with a yellow *G. fornicata* female, the modelled ‘perceptual distance’ between the two is 0.01 (unitless), which places them in the ca. fifth percentile of values recorded in the observational assay (Figure S1). When paired with a white spider morph, by contrast, this distance is increased to 0.26 units, which represents the ca. 75th percentile of ‘natural’ spider‐flower colour distances, as recorded in the observational assay. I used each replicate trio of inflorescences in only one trial (i.e. 1 day's observation), and replaced them at the start of each day.

To manipulate the physical proximity of flowers and spiders, I simply placed the dowel‐mounted inflorescences at a distance of either 60 cm beneath hub of a web in which a spider was resting (representing the 5th percentile of natural distances in the observational assay), or 430 cm from the hub of a web (representing the 95th percentile; Figure S1) in a haphazardly selected direction.

The complete set of four experimental treatments therefore comprised spiders paired with: (1) a colour matched, nearby flower, (2) a colour matched, distant flower, (3) a colour mis‐matched, nearby flower, and (4) a colour mis‐matched, distant flower. The design was fully balanced with *n* = 24 spiders per treatment, totalling 96 individuals. I randomised the assignment of treatments among spiders on each day, and sought to avoid re‐using individual spiders where possible by avoiding those in the same location on consecutive days. I did not mark individuals, however, and spiders did occasionally move short distances between days, so it is plausible that some individuals participated more than once. This should nonetheless contribute only random, residual variation to any observed effects. I otherwise estimated prey‐interception rates using the same walking transect‐based method described above.

### 2.3 Statistical analyses

For the observational assay, I used a general linear model to explore the relationship between hourly interception rates and the physical and perceptual proximity of spiders and flowers. I first constructed a global model with prey interception rate as the response, with physical distance as a fixed effect, colour distance and its second‐ and third‐order polynomials as fixed effects, and the interactions of all three colour distance terms and physical distance as interactions. My inclusion of colour distances as second‐ and third‐order polynomial terms was motivated by empirical demonstrations of both linearity and non‐linearity in colour discrimination (Fleishman et al., 2016; Garcia et al., 2017; Santiago et al., 2022). As applied to the current context, we might therefore expect any relationship between capture rates and spider‐flower colour distances to plateau as spiders become more similar to flowers, owing to pollinators' inability to effectively discriminate between the two at sufficiently small (‘threshold’) colour‐distances.

From this initial full model I conducted an information‐theoretic, AIC‐based process of model selection, by evaluating the relative information content of all possible subsets of this global model. As noted below I retained the leading candidate model in the set for inference, as indicated by the lowest AICc value, which included only physical and colour distance, and their interaction, as fixed effects. I favoured an information‐theoretic model‐selection approach here given the observational design of this assay, and the absence of a singular a priori expectation as to any relationship between capture success and physical and perceptual proximity to flowers.

Following the identification of a single leading model (see Section 3), I carried it through to the experimental assay in a formal hypothetico‐deductive test, and so included physical and colour distance, and their interaction, as fixed effects in my sole statistical model. I then used post‐hoc multiple comparisons on estimated marginal means to test for differences between all pairwise combinations of treatment levels. I visually inspected residual plots to validate model assumptions across all models. All statistical analyses were conducted in R (v4.2.1) with the ‘stats’ and ‘MuMIn’ (v1.47.1) packages (Bartoń, 2022; R Core Team, 2021).

### 2.4 Ethical note

No ethics permits were required for this work, and the only brief direct interaction with focal spiders (for spectral measurement) had no enduring ill‐effect on individuals. To the best of my knowledge, there was therefore no experimentally induced mortality, injury or lasting stress among study animals.

## 3 RESULTS

**FIGURE 2:**
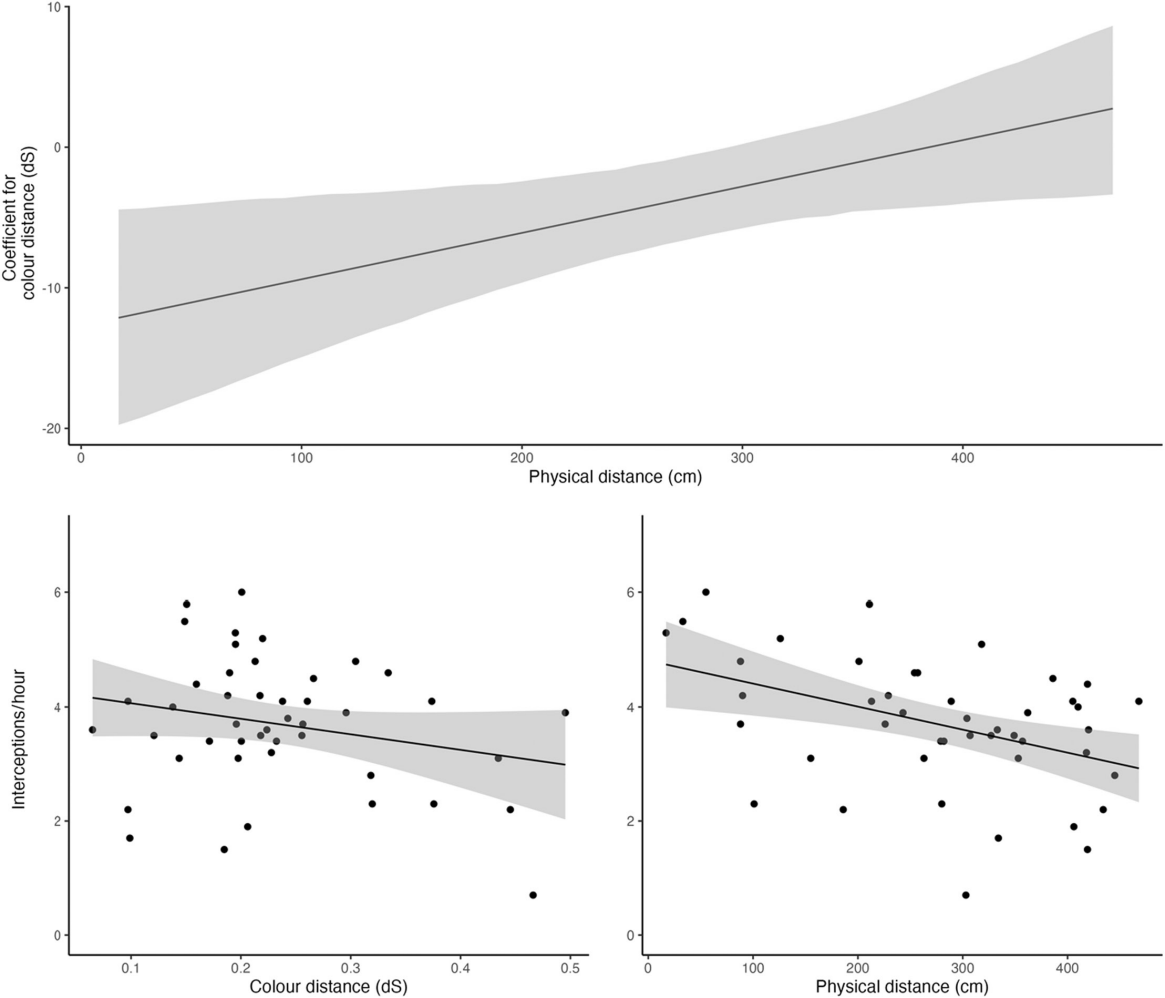
Results of the leading candidate general linear model which tested the relationship between prey capture success and the interaction of physical and perceptual proximity between spiders and flowers. The top panel shows how the *coefficient* for colour distance (not to be confused with colour distance itself) varies as a function of physical distance in a visual representation of the identified interaction, with negative coefficients representing smaller colour distances, or greater colour similarity, between spiders and flowers. That is, capture success becomes increasingly contingent on colour‐similarity between spiders and flowers as the two become physically proximate. Lower panels depict the main effects of colour and physical distance between spiders and flowers, alone.

In the observational assay, the most parsimonious model of prey interceptions (ΔAICc to next‐nearest model = 1.95, *w* = 0.239) included physical and colour distances, and their interaction, as fixed effects, and no higher‐order polynomial terms. The interaction was moderately strong (Table 1), and was characterised by a linear, negative effect of colour distance at closer physical distances (ca. 0–300 cm), which tapered to 0 as the proximity of spiders and flowers approached 400 cm (Figure 2). To state it in ecological terms, the visual similarity of spiders and flowers was increasingly predictive of prey capture success as the two became more physically proximate.

**TABLE 1:** Parameter estimates, their confidence intervals, unadjusted test statistics, and *p* values from a general linear model examining how the perceptual‐ and physical‐proximity between flowers and deceptive signalling spiders (*Gasteracantha fornicata*) influence the latter's rate of prey interception (interceptions per hour).

Parameter	Coefficient	SE	95% CI	*t*_41_	*p*
Intercept	7.85	1.00	5.89, 9.81	7.84	<.001
Physical proximity (cm)	−12.74	4.03	−20.64, −4.84	−3.16	.002
Perceptual proximity (unitless)	−0.01	0.01	−0.02, −0.005	−3.67	<.001
Physical × perceptual proximity	0.03	0.01	0.01, 0.06	2.45	.014

*Note*: Perceptual proximity was estimated as the continuous, Euclidean distance between spiders and their nearest inflorescence, as modelled in the colourspace of a representative Dipteran viewer. While physical distances are simply the straight‐line distance between spiders and the same inflorescence. The statistical model was the leading candidate among a broader set, as selected via an information‐theoretic procedure (see Section 2 for full details). *R* ^2^ = .367.

**FIGURE 3:**
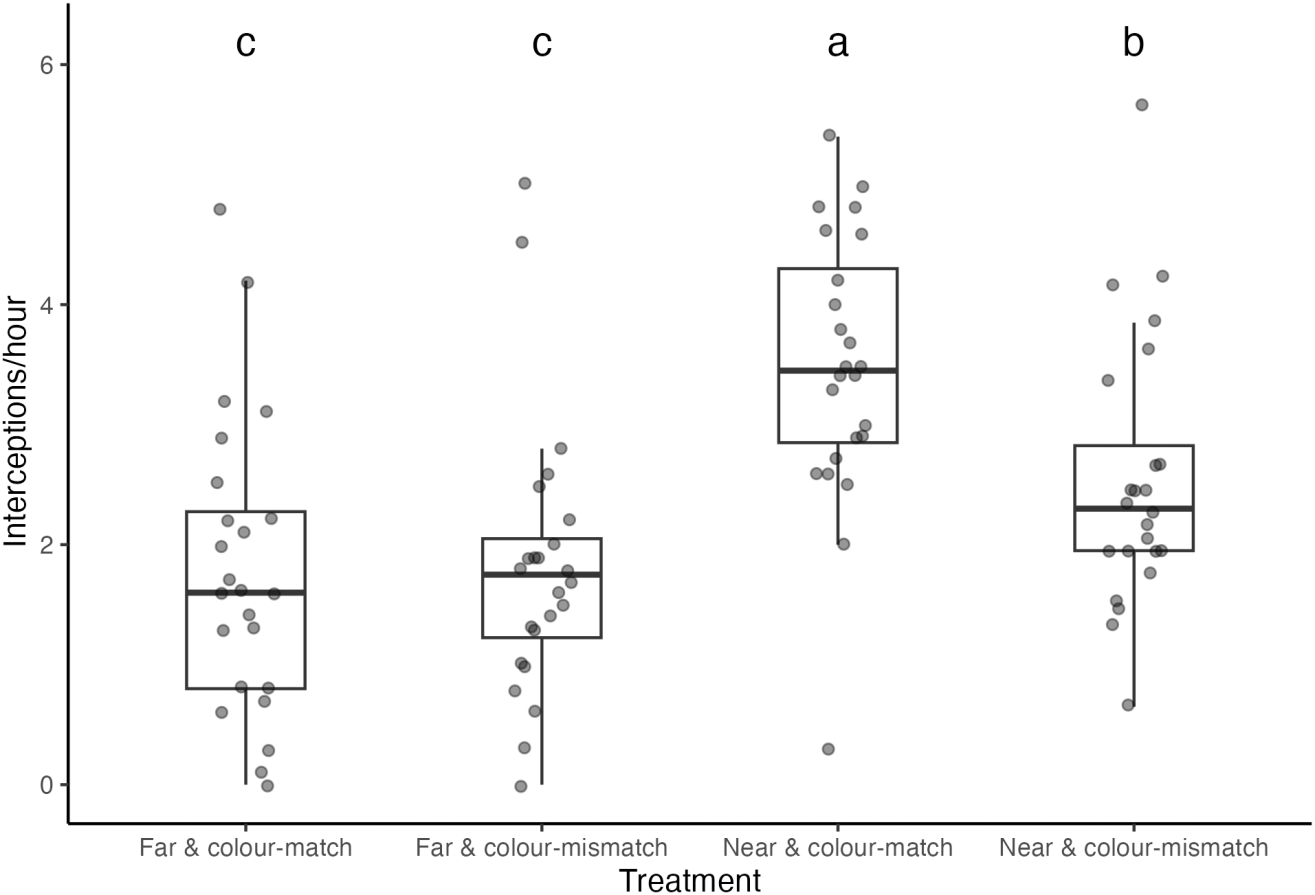
Results of the experimental assay which tested how the interaction of physical and sensory proximity between deceptive signalling spiders (*Gasteracantha fornicata*) and flowers shaped capture success. Signalling spiders (*n* = 24 per treatment group) were paired with inflorescences in a 2 × 2 factorial design to create the four treatments depicted: (1) distant, colour‐matched flowers (2) distant, colour‐mismatched flowers (3) nearby, colour‐matched flowers, and (4) nearby, colour mis‐matched flowers. Letters denote statistically significant differences between treatments identified from post‐hoc comparisons of estimated marginal means. General linear model *R* ^2^ = .272.

I was able to reconstruct this effect experimentally, with the manipulative assay revealing a weak but statistically significant interaction between the physical and perceptual proximity of spiders and flowers (Table 2). I found the highest capture rates among spiders which were paired with physically proximate & colour‐matched flowers (Figure 3). Spiders alongside colour‐mismatched flowers enjoyed the second‐highest rate of capture success, while interceptions were lowest and approximately equal among spiders paired with physically distant flowers, irrespective of their degree of colour‐matching. This too suggests a distinct, albeit modest, interactive effect of physical and perceptual proximity, with colour‐matching between spiders and local flowers being predictive of prey capture, albeit only when in the two are in close physical proximity.

**TABLE 2:** Parameter estimates, their confidence intervals, unadjusted test statistics, and *p* values from a general linear model examining the results of a manipulative test of how physical and perceptual proximity (via colour similarity) mediate the capture rates of the deceptive signalling jewelled spider *Gasteracantha fornicata*.

Parameter	Coefficient	SE	95% CI	*t*_92_	*p*
Intercept	1.79	0.24	1.33, 2.25	7.62	<.001
Physical proximity (near)	1.69	0.33	1.04, 2.34	5.07	<.001
Perceptual proximity (mis‐matched)	0.02	0.33	−0.64, 0.67	0.05	.960
Proximity (near) × perceptual (mis)	−0.96	0.47	−1.88, −0.04	−2.04	.042

*Note*: Signalling spiders were paired with inflorescences in a 2 × 2 factorial design and were fully balanced among treatment groups (*n* = 24). *R* ^2^ = .272.

## 4 DISCUSSION

Mis‐ and disinformation is rife in nature. Deceptive signals typically exploit cognitive and sensory biases in prey which are otherwise adaptive—such as colour preferences for foodstuffs—and theory predicts that the effectiveness of such signals should covary with their physical and/or perceptual proximity to the ‘models’ whose cues they are presenting (Ruxton et al., 2019). This dynamic is well established in Batesian mimicry (Mappes & Alatalo, 1997; Pfennig et al., 2001), but its application to aggressive contexts—where prey, their biases, and the models for deception are often more diverse—is poorly resolved. Here I used the generalist sit‐and‐wait predator *G. fornicata* to test this prediction. Across observational and experimental assays, I found that the ‘perceptual’ similarity of spiders and flowers interacted with their physical proximity to mediate their prey capture success. Rates of pollinator interceptions (and, hence, fitness) were elevated for spiders whose conspicuous colour signals most closely matched flowers, though only when the two were in close physical proximity (Figure 2). At greater physical distances this effect diminished, to a point where perceptual similar spiders/flower pairs had no advantage over dissimilar pairings (Figures 2 and 3). Together these results illuminate the importance of the broader information landscape in shaping the efficacy of deception or, conversely, receivers' vulnerability to misinformation. As discussed below, they also speak to open questions of sensory exploitation, and suggest adaptive solutions to sensory landscapes in flux.

The relatively short distance over which the interactive effect of colour‐ and physical‐distance operates offers some clue as to its mechanistic basis. Though the overall foraging ranges of many flies and bees are much larger than the distances examined here (Beekman & Ratnieks, 2000)—local exploration behaviour (Akter et al., 2017), short‐term memory (Menzel, 1982), recruitment dynamics (Dyer, 2002), floral constancy (Chittka et al., 1999) and visual adaption (Hempel de Ibarra et al., 2014) can operate at much finer spatial scales, and so may act alone or in concert to define the effects here observed. Arguably the simplest working explanation is that the combination of visual similarity and physical proximity leads flower‐visiting insects to mistake spiders for an inflorescence on the plant on which they are currently foraging. Flies and bees often use simple heuristics including colour, shape, orientation, and proximity to detect and classify the membership of inflorescences as belonging to a known‐rewarding plant or species (Hempel de Ibarra et al., 2014; Marden & Waddington, 1981). Pollinator foraging is thus fundamentally non‐random at small spatial scales. Indeed, constancy often increases with shorter inter‐inflorescence distances, with bees selectively ignoring closer, but unfamiliar, floral phenotypes in favour of those which resemble known rewards (Chittka et al., 1999; Marden & Waddington, 1981). In the current context, then, it seems likely that spiders who visually resemble co‐incident flowers may simply be mistaken for such by actively foraging pollinators, which is also consistent with recent evidence—both experimental and observational—for the mimicry of floral colour and shape in this system (White et al., 2017; White & Kemp, 2016a, 2016b). Conversely, sufficient colour difference and/or physical distance between flowers and spiders may render them recognisably distinct, giving rise to the interaction shown here (Figures 2 and 3). The absence of a spider‐less treatment in the current study leaves open the possibility that prey may also be ignoring spiders as non‐threatening, in addition to being actively attracted to spiders' signals. Previous, related studies in the system have included conceptually equivalent manipulations, however, and do strongly support active attraction as the more parsimonious explanation (Kemp et al., 2022).

While perceptual and physical proximity interactively shaped capture success, the latter was clearly the strongest single mediator of spider fitness. Co‐locating with flowers near doubled the capture success of spiders irrespective of their visual resemblance, while the benefit of colour‐matching flowers was only apparent for those spiders already in close proximity to flowers (Figures 2 and 3). That predators exploit resources desired by prey is well known (Heiling & Herberstein, 2004; White et al., 2022), though is noteworthy here in that it entails the aggressive mimicry of the resource itself (or at least the exploitation of shared cues; White et al., 2017; White & Kemp, 2020). A comparable effect has been shown only once previously in the flower‐mimicking orchid mantis (which, to human viewers, represents a more convincing mimic; O'Hanlon, Herberstein, & Holwell, 2014), and thus stands as a conceptual replication of the long‐hypothesised ‘magnet’ effect among deceptive predators (Annandale, 1900). ‘Magnet’ or ‘neighbourhood’ effects are those in which the attractiveness of flowers is shaped by the traits of their neighbours (Braun & Lortie, 2019; Peter & Johnson, 2008). They may be positive (increasing visitations for all) or negative (increasing visitations at the expense of neighbours) in direction and vary in magnitude depending on the density of neighbours and the traits used to signal to pollinators (Braun & Lortie, 2019). While such dynamics are clearly at work here (Figures 2 and 3), then, full reciprocal effects of spider and flower positioning on pollinator visitations remain to be resolved. Although predators such as *G. fornicata* reduce the pool of pollinators for neighbouring flowers, their often high densities (Kemp et al., 2013), attractiveness equivalent to or in excess of flowers (O'Hanlon, Holwell, & Herberstein, 2014), relatively low capture success (Uetz, 1992), and the frequent depletion of rewards even in profitable flowers (Corbet & Delfosse, 1984), means that the costs to their floral neighbours could be more than offset by an overall increase in pollinator visitations. In this case the spider‐flower relationship may be commensal or even mutualistic, which stands as a working hypothesis worthy of further study.

Sensory landscapes are in a constant state of flux, and with it the selective forces acting on organisms. Theory predicts both plastic and fixed solutions to this universal challenge (Calsbeek et al., 2012; De Jong, 1995), though the primary form of plasticity—behaviour—is more restricted in its scope for sit‐and‐wait predators such as *G. fornicata*. Given the benefits, as shown here, of physical and perceptual proximity to floral neighbours (Figures 2 and 3), selection should be intense to leverage this potential which, in the absence of behavioural flexibility, may instead favour maintenance of the polymorphism showcased by *G. fornicata* (among other deceptive signallers; White & Kemp, 2015). The relative weakness of the colour‐similarity effect (Tables 1 and 2) combined with the unpredictability of any spider's immediate floral neighbourhood may favour the maintenance of discrete morphs, which effectively represent ‘hedged bets’ as to the appearance of nearby flowers (as one of several selective processes potentially at play; White & Kemp, 2016a, 2016b). Further to this working hypothesis, recent work has shown that the appearance of white and yellow *G. fornicata* represent fitness optima (Kemp et al., 2022), which are near‐centrally located in the colour distributions of sympatric flora (White et al., 2017). These results thus speak to our burgeoning understanding of colour polymorphism evolution in deceptive contexts (Mokkonen & Lindstedt, 2016; White & Kemp, 2015, 2016a, 2016b), and with it the broader maintenance of genetic diversity (Svensson, 2017).

Communication is a ubiquitous feature of multicellular life, and deception plays a central role in the ecology and evolution of predator–prey interactions. Here I further illuminate an exemplar of aggressive deception, and show that the polymorphic lures of the jewelled spider do not function in isolation. As in human domains (Huang., 2021), the efficacy of deception is predicated on physical and perceptual proximity to the ‘truth’. How the distribution and appearance of models shapes the maintenance of deceptive polymorphism, the extent to which mimicry is harmful to models, and the mechanistic basis of deception over small spatial and temporal scales are compelling problems, for which tractable systems such as *G. fornicata* hold excellent promise.

## AUTHOR CONTRIBUTIONS

**Thomas E. White:** Conceptualization (equal); data curation (equal); formal analysis (equal); funding acquisition (equal); investigation (equal); methodology (equal); visualization (equal); writing – original draft (equal); writing – review and editing (equal).

## Supporting information


Figure S1.

## Data Availability

All data and code are available persistently archived via Zenodo (doi: 10.5281/zenodo.10701317).
