# Supplementary material for: Deceptive pollinator lures benefit from physical and perceptual proximity to flowers
Source: Ecol Evol. 2024 Mar 6;14(3):e11120. doi: 10.1002/ece3.11120 (PMC10917580; doi:10.1002/ece3.11120)
Supplement: Supplementary file 1 — Figure S1. [file ECE3-14-e11120-s001.pdf]

Supplementary material for: Deceptive pollinator lures benefit from physical and perceptual proximity to flowers

Supplementary figures

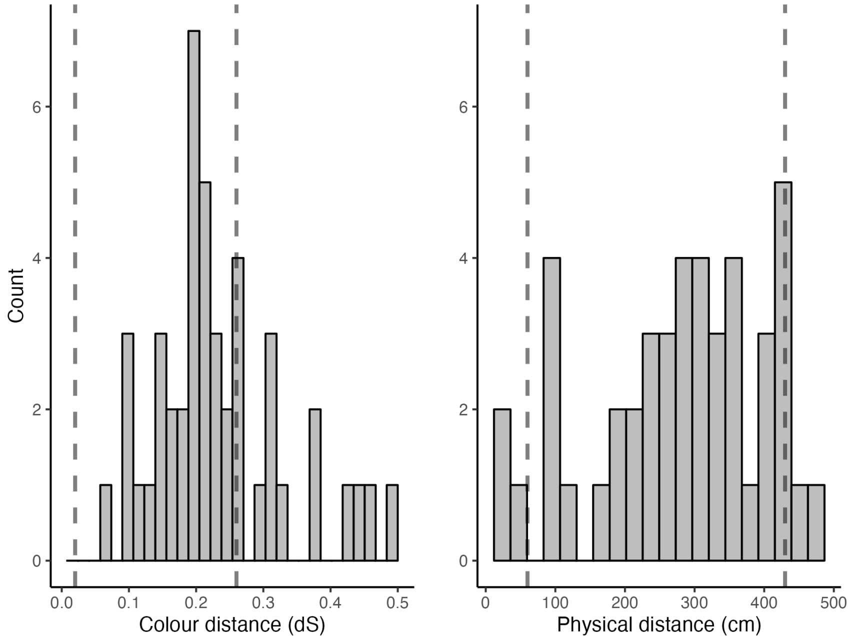

Figure S1: Distributions of the colour- (left) and physical- (right) distances between deceptive signalling *Gasteracantha fornicata* and the nearest inflorescence in the observational assay. Dashed vertical lines denote the values of the four treatments achieved in the subsequent manipulative experiment.
